# Structural Characterization of Minor Ampullate Spidroin Domains and Their Distinct Roles in Fibroin Solubility and Fiber Formation

**DOI:** 10.1371/journal.pone.0056142

**Published:** 2013-02-13

**Authors:** Zhenwei Gao, Zhi Lin, Weidong Huang, Chong Cheong Lai, Jing-song Fan, Daiwen Yang

**Affiliations:** Department of Biological Sciences, National University of Singapore, Singapore; Massachusetts Institute of Technology, United States of America

## Abstract

Spider silk is protein fibers with extraordinary mechanical properties. Up to now, it is still poorly understood how silk proteins are kept in a soluble form before spinning into fibers and how the protein molecules are aligned orderly to form fibers. Minor ampullate spidroin is one of the seven types of silk proteins, which consists of four types of domains: N-terminal domain, C-terminal domain (CTD), repetitive domain (RP) and linker domain (LK). Here we report the tertiary structure of CTD and secondary structures of RP and LK in aqueous solution, and their roles in protein stability, solubility and fiber formation. The stability and solubility of individual domains are dramatically different and can be explained by their distinct structures. For the tri-domain miniature fibroin, RP-LK-CTD_Mi_, the three domains have no or weak interactions with one another at low protein concentrations (<1 mg/ml). The CTD in RP-LK-CTD_Mi_ is very stable and soluble, but it cannot stabilize the entire protein against chemical and thermal denaturation while it can keep the entire tri-domain in a highly water-soluble state. In the presence of shear force, protein aggregation is greatly accelerated and the aggregation rate is determined by the stability of folded domains and solubility of the disordered domains. Only the tri-domain RP-LK-CTD_Mi_ could form silk-like fibers, indicating that all three domains play distinct roles in fiber formation: LK as a nucleation site for assembly of protein molecules, RP for assistance of the assembly and CTD for regulating alignment of the assembled molecules.

## Introduction

Spider silk is an ideal super material due to its extraordinary mechanical properties compared to other available materials [1]–[3]. However, mass production of spider silk from nature is still impossible because the farming of spiders is limited by their cannibalistic and territorial behavior [4]. Thus, producing spider silk by recombinant biotechnology is one of the most promising alternatives [3], [5]–[7]. Before achieving this goal, one needs to understand the molecular structures, self-assembly mechanism and fiber formation of spider silk proteins, which are affected by solvent environment and shear and elongational forces [3]. Female orb-weaving spiders can produce up to seven types of silk with different mechanical properties by using different types of silk proteins [2]. Until now, complete gene sequences have been known for only dragline silk (or major ampullate silk) from black widow spider (*Latrodectus hesperus*) [8], and only partial gene sequences have been determined for other spider silks. According to the known spider silk protein sequences, silk proteins share several common features in primary structures: (i) large number of amino acids, (ii) many highly repetitive units flanked by non-repetitive N-terminal domain (NTD) and C-terminal domain (CTD), and (iii) high abundance in Ala, Gly or/and Ser. Each repetitive unit of major ampullate spidroin (MaSp) contains multiple repeats of short simple motifs such as (A)_n_ (n = 4–10) and GGX (X = A, Q or Y) [2], [8]. Similarly, the repetitive unit of minor ampullate spidroin (MiSp) also has repeats of such short motifs [9]. Differently, each MiSp repetitive unit contains an additional relatively large domain that lacks repeats of short motifs [9], [10]. Different from MaSp and MiSp, the repetitive units of aciniform and tubuliform silk proteins are complex and lack of the short motifs [11]–[15]. At present it is clear that the composition of the repetitive units varies from one type of silk protein to another, which is suggested to determine the mechanical properties of a given type of silk.

Unlike the repetitive units, CTDs are relatively conserved among all spider silk proteins except the web glue proteins and are assumed to perform the same function [16]. This also applies to NTDs [17]. Recent studies on two spider species have shown that the NTD of MaSp can regulate the self-assembly of silk proteins in a pH dependent manner [18] and the CTD of MaSp can prevent premature aggregation by stabilizing the solution state of silk proteins and direct the alignment of repetitive units to form well defined fibers [19]–[21]. In spite of the high sequence identity (58%) between the CTDs of MaSp (CTD_Ma_) from *A. diadematus* and *E. australis*, these two domains have distinct biophysical properties. For example, the intermolecular disulfide bridge in the CTD_Ma_ from *A. diadematus* was considered to be important to the domain stability [21], but the disulfide bridge was found to have only slight contribution to the thermal stability of the domain from *E. australis* and to be not critical for fiber formation [19]. The CTD sequence identity among different types of silk proteins is lower than that among the same type of proteins from different species. For instance, the CTD of tubuliform spidroin (TuSp) and CTD_Ma_ from the same *N. antipodiana* share 29% sequence identity. Due to the low sequence identity, the CTD_Ma_ existed as a dimer [21] but the isolated CTD of TuSp (CTD_Tu_) existed as oligomers in aqueous solution [22]. Therefore, the same type of CTDs from different species and different types of CTDs from the same species may display diverse biophysical properties. In order to demonstrate whether different CTDs perform the functions in the same or different molecular mechanisms, it is necessary to characterize the structures and biophysical properties of individual domains of different silk proteins and their functional roles in silk formation and protein storage.

**Figure 1:**
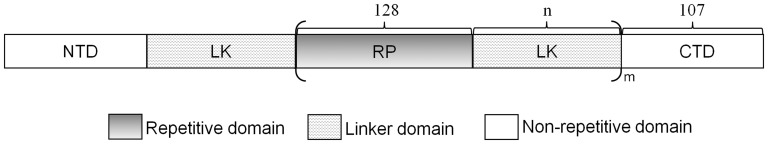
Molecular organization of MiSp. The amino acid (aa) number of each domain is indicated above the corresponding bar, if it is available. The number of aa in LK domain (n) varies from 83–174. The total number of RP and LK in the MiSp sequence is still unknown and is denoted as “m”. The sequences of the NTD and its adjacent LK domain are unknown.

We previously reported a MiSp clone, clone 145, from the total silk gland cDNA library of *N. antipodiana* [12]. The deduced amino acid (aa) sequence comprises one repetitive domain (RP_Mi_, 128aa, previously named as spacer), one non-repetitive C-terminal domain (CTD_Mi_, 107aa), and one linker domain (LK_Mi_, 89aa, previously named as repetitive sequence) that links RP_Mi_ and CTD_Mi_ or in general links two structured domains (Figure 1). Until now, the N-terminal domain sequence has not been determined yet for any MiSps. RP_Mi_ and CTD_Mi_ are conserved among different spider species (Figure S1), but LK_Mi_s vary significantly in number of amino acids among different repetitive units in the same MiSp [9], [10]. Although the CTD_Mi_ from *N. antipodiana* and CTD_Ma_ from *A. diadematus* share 44% sequence identity, the CTD_Mi_ contains no cysteine residues but the CTD_Ma_ has one disulfide linkage between two molecules which can enhance the stability of CTD_Ma_ [19], [21]. Moreover, RP_Mi_ is unique to MiSps and its functional roles in protein storage and fiber formation are unknown. Besides the difference in amino acid sequences, MaSp silk is elastic when stretched and MiSp displays irreversible deforming [9]. Thus, MiSp may adopt a different self-assembly and fiber formation mechanism than the well characterized MaSp. In this work, we report the three-dimensional (3D) structure of CTD_Mi_ from *N. antipodiana*, the secondary structures of RP_Mi_ and LK_Mi_ and their roles in conferring protein stability, solubility and fiber formation.

## Materials and Methods

### Cloning of RP and LK Domains of MiSp from *N. antipodiana*

Forward (5′-gcaaatgctatgaacagtttacttggt-3′) and reverse (5′-attgcctaatgttgatacatatccacta-3′) primers were designed on the basis of the known sequence of RP_Mi_ [12]. MiSp fragments each containing one LK domain flanked by partial RP_Mi_ sequences were obtained by polymerase chain reaction (PCR) from genomic DNA.

### Protein Sample Preparation

The DNA sequence of our previously identified MiSp fragment (clone 145) [12] was confirmed here by PCR from our spider genomic DNA. The target genes encoding different MiSp regions (CTD_Mi_, RP_Mi_, RP-LK_Mi_, LK-CTD_Mi_, RP-LK-CTD_Mi_) were amplified from clone 145 using specific primers and subcloned into a pET32-derived expression vector. The recombinant plasmids were transformed into *E. coli* BL21 strain (DE3). Cells were grown in LB or M9 medium at 37°C to an OD_600_ of 0.6. Right after induction by 0.2 mM IPTG (isopropyl β-D-thiogalactoside), cells were shifted to 20°C and further cultured for 16 hrs. For ^13^C,^15^N-labeled (^15^N-labeled) samples, the cells were cultured in M9 medium which contained only ^15^N-labeled NH_4_Cl and ^13^C-labeled (non-labeled) D-glucose as the sole nitrogen and carbon source. After over-expressed, the proteins were purified by immobilized metal affinity chromatography, gel filtration and then ion exchange columns. All the proteins used here contained a 6xHis-tag and a thrombin cleavage sequence at the N-terminus.

### NMR Spectroscopy

All NMR experiments were performed on a Bruker 800 MHz NMR spectrometer at 25°C. Non-labeled CTD_Mi_ (∼0.6 mM), RP_Mi_ (∼0.6 mM) and RP-LK-CTD_Mi_ (∼0.6 mM and ∼3 mM) in 10 mM phosphate buffer (pH 6.8) were used to acquire one-dimensional (1D) ^1^H NMR spectra. The 1D NMR spectra were recorded using the water gate W5 pulse scheme with 64 scans and an interscan delay of 2 s. ^15^N-labeled CTD_Mi_ (∼0.5 mM) and RP_Mi_ (∼0.5 mM) in 10 mM phosphate buffer were employed to record 2D ^1^H-^15^N HSQC spectra. The samples used for structure determination of CTD_Mi_ contained 1 mM ^13^C,^15^N-labeled protein, 10 mM phosphate buffer (pH 6.8), 5 mM EDTA, 50 mM NaCl and 0.01% sodium azide. To obtain sequence-specific assignments and nuclear Overhauser effects (NOEs), the following spectra of CTD_Mi_ were recorded: 2D ^1^H-^15^N HSQC, 2D ^1^H-^13^C HSQC, 3D HNCA, 3D HN(CO)CA, 3D MQ-CCH-TOCSY[23], 4D time-shared^ 13^C, ^15^N-edited NOESY [24]. Inter-molecular NOEs were identified from a ^13^C,^15^N-filtered 3D experiment on a sample containing 50% ^13^C,^15^N-labeled and 50% unlabeled proteins [25]. This sample was prepared by mixing equal amount of labeled and unlabeled proteins in 8 M urea for 2 hrs and then removing the urea by dialysis against 10mM phosphate buffer. All the spectra were processed by NMRpipe and analyzed by following the strategy described previously [26] and using NMRspy and XYZ4D (http://yangdw.science.nus.edu.sg/Software&Scripts/XYZ4D/index.htm). CYANA [27] was employed for structure calculation. 10 dimer structures with the lowest final target function values were chosen from 100 calculated ones. PROCHECK-NMR [28] was used to evaluate the quality of the structures. Protein structure validation software (PSVS) [29] (http://psvs-1_4-dev.nesg.org/) was utilized to assess the all-atom steric clashes [30].

### Circular Dichroism and Protein Unfolding

All circular dichroism **(**CD) spectra were recorded on a Jasco J-810 spectropolarimeter equipped with a thermal controller. A 0.1 cm path length cuvette was used for all CD experiments. The far-UV spectrum of RP_Mi_ was recorded using a 20 µM protein in 10 mM sodium phosphate at pH 6.8. Both urea- and thermal-induced unfolding processes were monitored at 222 nm using samples with 10 µM protein, 10 mM sodium phosphate at pH 6.8. Except for RP-LK-CTD_Mi_, urea denaturation curves for other MiSp constructs were analyzed with the following equation derived from a two-state unfolding model [31].
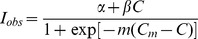
(1)where *I_obs_* is the experimental signal intensity in the presence of *C* molar urea, *α* and *β* the intercept and slope of the pre-transition zone respectively, *C_m_* is the urea concentration at the transition midpoint, and *m* is the slope at the transition midpoint. For urea-denaturation of RP-LK-CTD_Mi_, the experimental data were fitted using a linear combination of two two-state unfolding equations. Eq. 1 was also used to obtain *T_m_* by replacing *C* and *C_m_* by *T* and *T_m_*, respectively, where *T* is temperature, and *T_m_* is the temperature at the transition midpoint.

### Size Exclusion Chromatography

A Superdex™ 75 PG (GE Healthcare) column with a total volume of 120 ml was used to run all the protein samples. The running buffer for RP_Mi_ contained 10 mM sodium phosphate (pH 6.8) with or without 100 mM NaCl. For other samples, only 10 mM sodium phosphate at pH 6.8 was used. The flow rate used was 1 ml/min, and fractions were collected every 2 ml. The fractions were analyzed by SDS-PAGE to confirm which peak in the UV absorbing profile corresponded to the target protein. A molecular mass standard set consisting of Ribomuclease A (13.7 kDa), Chymotrypsinogen A (25 kDa), Ovalbumin (43 kDa), and BSA (67 kDa) was chromatographed to estimate the apparent molecular weights of target proteins.

### Protein Solubility

The purified protein samples in respective 10 mM sodium phosphate and 10 mM Tris buffers (pH 7.0) were concentrated using centrifugal filter units with 3 kDa cutoff membrane at centrifugal force of 3000×g. When the protein concentration was >5 mg/ml, 2 µl samples were regularly taken out from the solution until precipitate or gel was observed. Otherwise, larger volumes of samples were taken for concentration measurements. To determine protein concentrations, the samples taken were diluted in the same buffers as those used for the protein samples. The concentrations were measured using the absorbance at 280 nm and also estimated using SDS PAGE.

### Shear Force-Induced Aggregation

To study protein aggregation induced by shear force that plays a critical role in the natural silk spinning process, samples of 2 ml with 0.05 mg/ml proteins and 10 mM phosphate buffer (pH 6.8) were placed into a UV/Vis cuvette with a small magnetic star bar stirring at 500 rpm, 25°C. The turbidity of the samples was monitored by measuring OD_350_ on a BIO-RAD Smart Spec™ Plus Spectrophotometer at a series of time intervals.

To determine the effect of sodium chloride and sodium phosphate on the aggregation of RP-LK-CTD_Mi_, shear force-induced aggregation experiments were performed under two salt concentrations: 0 and 200 mM. The samples (1 mg/ml) placed in a 2 ml eppendorf tube were shaken at 150 rpm, 25°C in an incubation shaker. At different time points, the samples were taken out. After removing the precipitate by centrifuge, the concentration of the soluble portion was measured and then the total amount of precipitated protein was calculated.

### Scanning Electron Microscopy

1 ml purified protein sample containing 5 mg/ml RP-LK-CTD_Mi_ in 10 mM sodium phosphate buffer (pH 6.8) was placed into a 2 ml eppendorf tube and the sample was shaken at 200 rpm, 25°C for 5 minutes in an incubation shaker. Then, silk-like fibers formed in the tube were picked out by a needle. SEM micrographs of the fibers were observed on a JEOL JSM-6510 and photographed at a voltage of 15 kV and room temperature (24–26°C).

### Prediction of Disorder, Hydrophobicity and Aggregation Propensity

The disordered residues in LK_Mi_ were predicted using PONDR-FIT (http://www.disprot.org/pondr-fit.php). If the disordered score of a residue is >0.5, this residue is considered as disordered [32]. The aggregation-prone regions in LK_Mi_ were predicted using Zyggregator (http://www-vendruscolo.ch.cam.ac.uk/zyggregator.php). When a region of several consecutive residues each have aggregation scores larger than 1, this region is considered to be prone to aggregate [33]. The hydrophobicity plot of LK_Mi_ was obtained using Protscale (http://web.expasy.org/cgi-bin/protscale/protscale.pl) with the scale option of Hphob./Roseman [34].

## Results and Discussion

### Sequences of RP and LK Domains

Our PCR results from the genomic DNA show that all the repetitive domains in the MiSp from *N. antipodiana* are identical. At present, the exact number of repeats has not been determined yet because the repetitive feature of the RP_Mi_ in DNA. We identified 5 types of linker domains with different size ranging from 83 to 174 aa in genomic DNA (Figure S2). Glycine (45–48%) and alanine (33–39%) are dominant in linker domains, which are consistent with previous reports [9], [10]. RP_Mi_ is highly conserved among different species (Figure S1B). Interestingly, the linker domain between the CTD and RP domains of *N. antipodiana* (LK_Mi_) obtained here is much shorter than that of *N. clavipes* [9].

### Solution Structures of CTD_Mi_, RP_Mi_ and LK_Mi_

**Figure 2:**
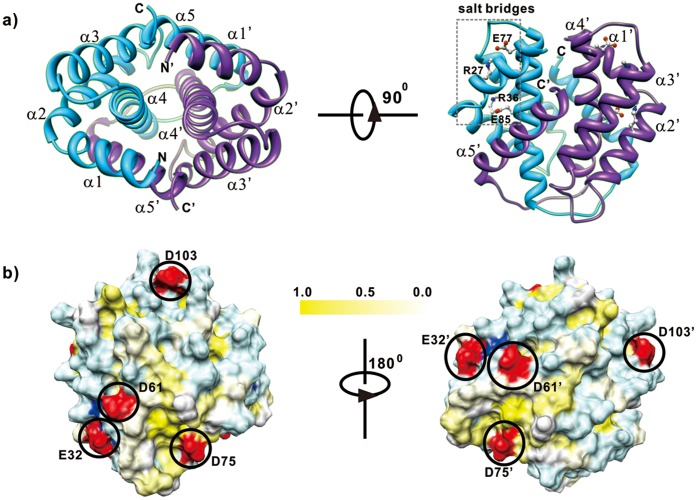
Structure and surface plots of CTD. a) Cartoon drawing of the lowest energy conformer of CTD. The two monomers are indicated by two different colors. b). Hydrophobic and charged surface of CTD. Hydrophobic residues are colored by a scale based on normalized hydrophobicity values [34]: Phe (1.0) for yellow, Val (0.57) for light yellow and Gly (0.0) for white. Positively charged, negatively charged and polar residues are colored by blue, red and light blue.

In aqueous solution, CTD_Mi_ formed a stable homodimer as evidenced by size exclusion chromatography (SEC, Figure S3). The structure of CTD_Mi_ was determined using distance and dihedral restraints derived from multidimensional NMR spectroscopy (Figure 2A and Table 1). Overall, the structure of CTD_Mi_ adopts a globular fold of two twisted five-helix bundles (α1 [Gly^18^-Leu^28^], α2 [Ala^31^-Val^45^], α3 [Leu^55^-Ser^69^], α4 [Asp^75^-Ser^97^], α5 [Val^107^-Met^122^] which pack in parallel to form a homodimer. α5 is swapped to stabilize the dimeric structure. The major dimer interface involves helices α1/α5’, α4/α4’ and α5/α1’. Many hydrophobic residues are located in the interface and are in close contact, suggesting that hydrophobic interactions are the dominant factor for holding the two monomers together. Similarly, hydrophobic interactions among different helices in each monomer (involving 26 hydrophobic residues) are critical for the stability of the monomer. In addition, α4 is connected with α1 and α2 through two salt bridges R27-E77 and R36-E85 in each monomer. The formation of the R36-E85 salt bridge is evident from the extremely large chemical shift of ^1^H_ε_ of R36 (11.7 ppm) and the observation of ^1^H_η1_ (10.5 ppm) and ^2^H_η2_ (5.8 ppm) of R36. Although ^1^H_η1_ and ^2^H_η2_ of R27 were not detected, the side-chains of R27 and E77 are in close proximity to be able to form a salt-bridge. Mutation of R27 into A27 reduced the transition temperature of thermal denaturation (*T_m_*) by ∼20°C (Figure S4), confirming the presence of the R27-E77 salt bridge.

**Table 1:** Experimental restraints and structural statistics for ten lowest-energy NMR structures out of 100 calculated structures.

NMR distance constraints	
Total intra-molecular NOE	2697
Intra-residue	899
Sequential	736
Medium-range (1<|I-j|<5)	760
Long-range (|I-j|≥5)	302
Inter-molecular NOE	171
Dihedral restraints*	116
Structural statistics	
Violation	mean±SD
Distance violation (Å)	0.42±0.06
Dihedral angle violation (°)	3.46±0.58
Max. dihedral angle violation (°)	4.64
Max. distance constraint violation (Å)	0.49
Ramachandran plot region (all residues) [%]	
most favored	90.1
additionally allowed	9.3
generously allowed	0.3
disallowed	0.3
Mean RMS deviation from the average coordinates (Å)†	
Backbone atoms (C^α^, C’, N, O)	0.50±0.14
All heavy atoms	0.82±0.11
MolProbity clash score	−6.48

* Dihedral angle constraints were generated by TALOS based on C_α_ and C_β_ chemical shifts.

† Average rms deviation in the structural region (residue 17–123) was calculated among 10 refined structures.

The overall structure of CTD_Mi_ is very similar to the previously reported structure of CTD_Ma_ [21] with a Dali Z-score of 15. In addition, both CTD_Mi_ and CTD_Ma_ contain two intra-molecular salt bridges and have many hydrophilic residues located on the surface. Nevertheless, there are several key differences in local structures. 1) For CTD_Mi_ dimer, eight negatively charged carboxyl groups (four in each monomeric unit: E32, D61, D75, D103) are exposed on the protein surface (Figure 2B), but no net charges on the surface of CTD_Ma_ (Figure S5). Note that each CTD_Ma_ monomer contains only two negatively charged carboxyl groups and two positively guanidinium groups which form two salt bridges [21] and are buried. 2) CTD_Mi_ contains no cysteine residues and there is no intermolecular disulfide bridge, but one intermolecular disulfide bond exists in the CTD_Ma_ dimer [21]. 3) There are more hydrophobic residues located in between α5 and α3 and between α5 and α1’ in CTD_Mi_ than in CTD_Ma_ (Figure S6).

**Figure 3:**
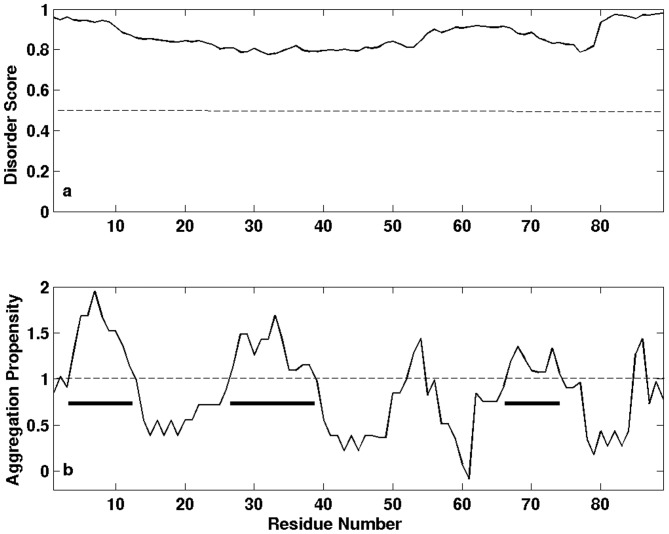
Predicted disordered residues (a) and aggregation regions (b) in LK_Mi_. Disordered residues have scores >0.5. The aggregation-prone regions with aggregation propensity scores >1 are indicated by bars in panel b.

**Figure 4:**
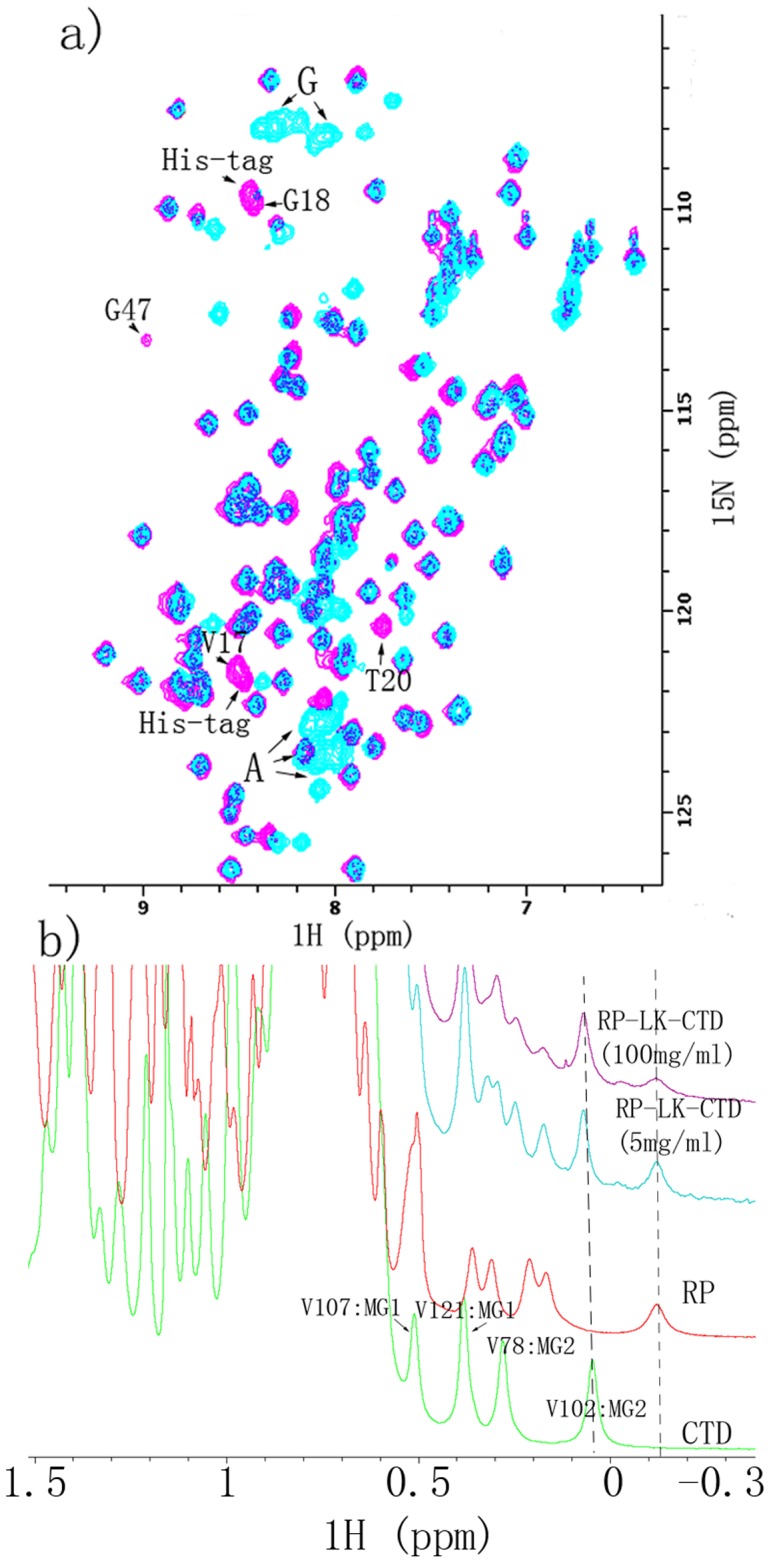
NMR spectra of LK-CTD_Mi_, CTD_Mi_, RP_Mi_ and RP-LK-CTD_Mi_. a). Overlay of 2D ^1^H-^15^N HSQC spectra of isolated CTD_Mi_ (red) and di-domain LK-CTD_Mi_ (cyan). The signals from the His-tag in the isolated CTD_Mi_ were labeled. Correlation peaks from Ala and Gly of the LK domain in LK-CTD_Mi_ are clustered in small regions and are indicated by arrows. b). stacked plot of 1D ^1^H spectra (−0.5 ppm –1.6 ppm) of CTD_Mi_ (green, 0.6 mM), RP_Mi_ (red, 0.6 mM), RP-LK-CTD_Mi_ (cyan, 0.6 mM) and RP-LK-CTD_Mi_ (pink, 3 mM).

LK_Mi_ (89 aa) contains 46.1% Gly and 32.3% Ala (Figure S7A). It was predicted to be intrinsically disordered (Figure 3A). Except the region of G54-Y70, most residues have hydrophobic scores larger than zero (Figure S7B), implying that LK_Mi_ has low water solubility. To determine experimentally the secondary structure of LK_Mi_, we tried to produce it in *E. coli*, but the production was not successful because it was degraded rapidly during the purification process. Thus we used the bi-domain fragment, LK-CTD_Mi_. A comparison of the ^1^H-^15^N HSQC spectra of the LK-CTD_Mi_ and CTD_Mi_ reveals that the backbone ^1^H-^15^N correlation peaks for the residues from the LK domain are located in the range of 7.7–8.5 ppm in the ^1^H dimension and most Gly and Ala ^1^H-^15^N correlations are clustered together (Figure 4A). This result shows that the LK domain is indeed intrinsically disordered. Except the correlation peaks from the N-terminal region of the isolated CTD_Mi_ (e.g., V17, G18 and T20), other peaks from the isolated CTD_Mi_ have the same ^1^H and ^15^N chemical shifts as those from the CTD in the bi-domain LK-CTD_Mi_. Note that V17 is the N-terminal end residue of the CTD domain and is the connection site of the LK and CTD in the LK-CTD_Mi_ construct. The signal of G48 in the bi-domain was weak and had the same chemical shifts as the G48 in the isolated CTD although it is not visible in Figure 4A. The results indicate that there are no or only weak interactions between the two domains at a protein concentration equal to or less than 0.5 mM. To evaluate the aggregation propensity of the LK_Mi_, we predicted the aggregation-prone regions. The prediction shows that LK_Mi_ contains three aggregation-prone regions with aggregation propensity scores >1: Y4-A12, A27-A37 and G67-A74 (Figure 3B).

**Figure 5:**
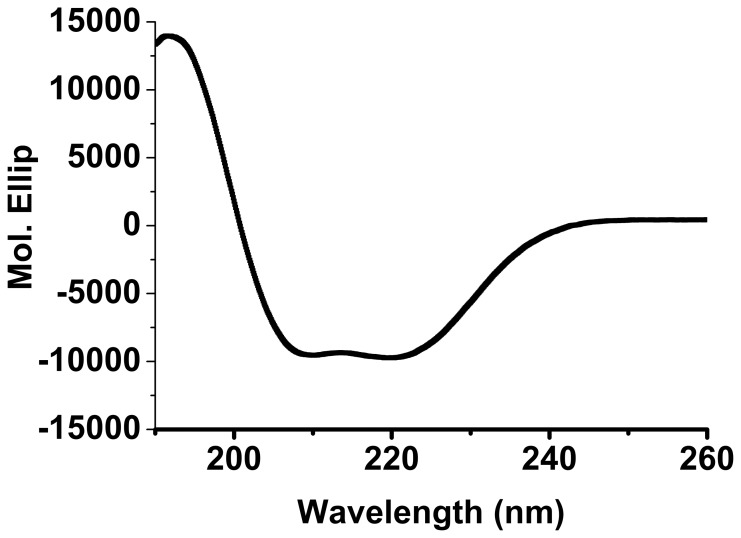
Circular Dichroism (CD) spectrum of RP_Mi_.

RP_Mi_ was folded into mainly α-helical structure in water as shown by its circular dichroism (CD) spectrum (Figure 5). It existed mainly in a monomeric form at low protein concentration (<1 mg/ml) in the presence of 100 mM NaCl, but mainly in a dimer together with small oligomers in the absence of salt as indicated by SEC (Figure S3). Very likely, the monomer, dimer and larger oligomers are in dynamic equilibrium since a single SEC peak was observed. The 1D ^1^H NMR spectrum of RP_Mi_ shows that this domain adopts a folded 3D structure since its methyl proton signals display good dispersion with one methyl at −0.13 ppm (Figure 4B). The line width of the peak at −0.13 ppm was 40 Hz, which was significantly larger than that for the methyl signal of CTD_Mi_ at 0.03 ppm (26 Hz) (Figure 4B). Because the NMR line width is proportional to the molecular size, the apparent size of RP_Mi_ must be significantly larger than the size of CTD_Mi_ dimer under the condition of 0.6 mM protein, 10 mM sodium phosphate and pH 6.8. As mentioned earlier, RP_Mi_ (128 aa) consists of only ∼20% more aa than CTD_Mi_ (107 aa). Therefore, the NMR result further shows that RP_Mi_ should exist in equilibrium between dimers and small oligomers at low salt concentration (10 mM sodium phosphate) and relatively high protein concentration (0.6 mM). Due to the oligomerization-prone feature, we have not solved RP_Mi_’s 3D structure yet.

At low protein concentrations (<1 mg/ml), RP-LK-CTD_Mi_ existed as a dimer on the basis of the SEC data (Figure S3). The dimer should be mediated through the CTD domain since the dimerization of CTD_Mi_ is independent of protein concentration. A comparison of the 1D ^1^H NMR spectra of RP_Mi_, CTD_Mi_ and RP-LK-CTD_Mi_ (Figure 4B) reveals that no strong interactions exist among the three different domains in RP-LK-CTD_Mi_ because the isolated methyl signals from the tri-domain protein have nearly the same chemical shifts as those in the isolated individual domains. The line width of the methyl signals at 0.03 ppm and −0.13 ppm in the isolated CTD_Mi_ and RP_Mi_ were 26 Hz and 40 Hz respectively, very similar to those of the tri-domain fragment (27 Hz and 42 Hz) at a concentration of 0.6 mM, although the tri-domain is almost three times larger than the individual CTD_Mi_ and RP_Mi_ in molecular weight. This can be explained by the independent motions of the CTD_Mi_ dimeric unit and RP_Mi_ domain due to the high flexibility of the disordered linker domain. When the RP-LK-CTD_Mi_ concentration was increased from ∼0.6 mM to ∼3 mM, the line width of the signal at 0.03 ppm (from the CTD) increased slightly from 27 Hz to 30 Hz, while the line width of the signal at −0.13 ppm (from the RP domain) increased dramatically from 42 Hz to 90 Hz. The result indicates that the tri-domain molecules assemble together to form oligomers through the weak association of RP and LK domains from different molecules.

### Stability of CTD_Mi_, RP_Mi_, LK-CTD_Mi_ and RP-LK-CTD_Mi_

**Figure 6:**
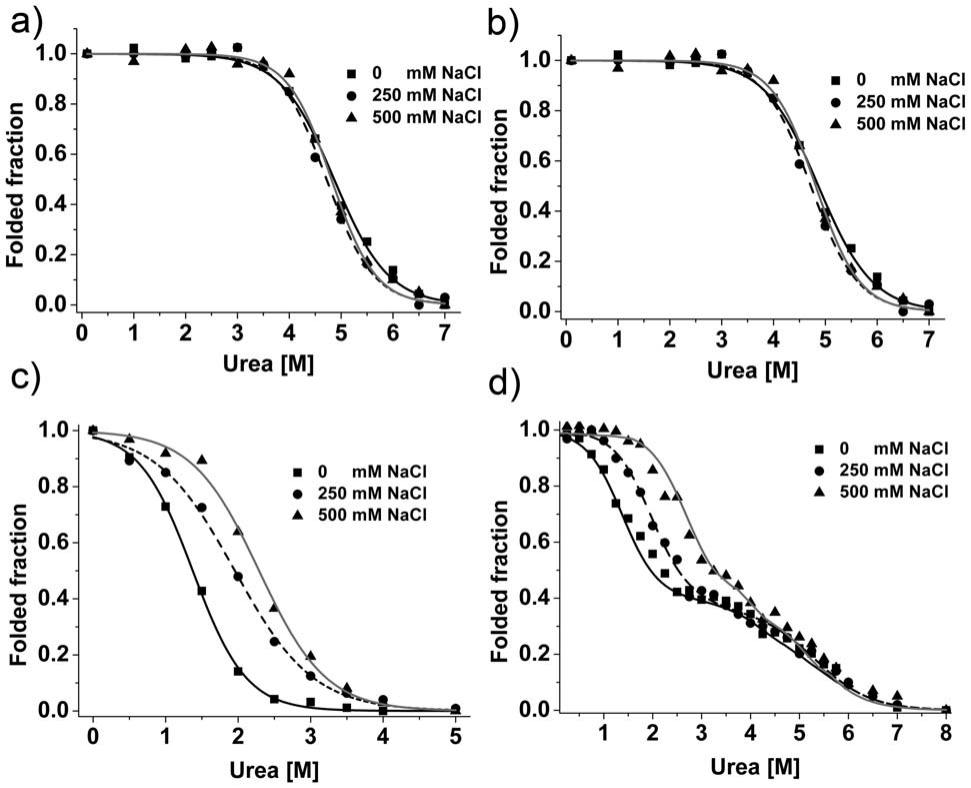
Urea-induced denaturation of MiSp fragments under different NaCl concentrations monitored by CD. a) CTD_Mi_; b) LK-CTD_Mi_; c) RP_Mi_; d) RP-LK-CTD_Mi_. The lines represent the fitting curves. All the data were normalized.

Full length silk proteins are extremely water soluble and stable when stored in the silk glands [35]. To understand how silk proteins are stored stably at high concentration, we investigated the stability and solubility of individual protein domains and their dependences on salt and protein concentrations that change significantly when the proteins pass through the spinning duct [3]. Although CTD_Ma_ and CTD_Mi_ have similar overall structures, their chemical and thermal stabilities are significantly different. The transition midpoints in urea (*C_m_*) and temperature (*T_m_*) denaturation of CTD_Mi_ were ∼4.8 M urea and ∼71°C, respectively (Figure 6A and Figure S4, blue line), which are significantly larger than those of CTD_Ma_ (∼2 M urea at 10 mM phosphate and ∼2.8 M urea at 500 mM NaCl, 64°C at 10 mM phosphate) [21]. The result indicates CTD_Mi_ is much more stable than CTD_Ma_. Interestingly, NaCl had nearly no effect on the chemical stability of CTD_Mi_ (Figure 6A), while NaCl could stabilize CTD_Ma_ [21]. The stability of CTD_Mi_ was independent of protein concentration when the concentration was below 0.2 mM, but CTD_Ma_ was much more stable against urea denaturation at a protein concentration of 5 µM than 0.2 mM [21].

**Figure 7:**
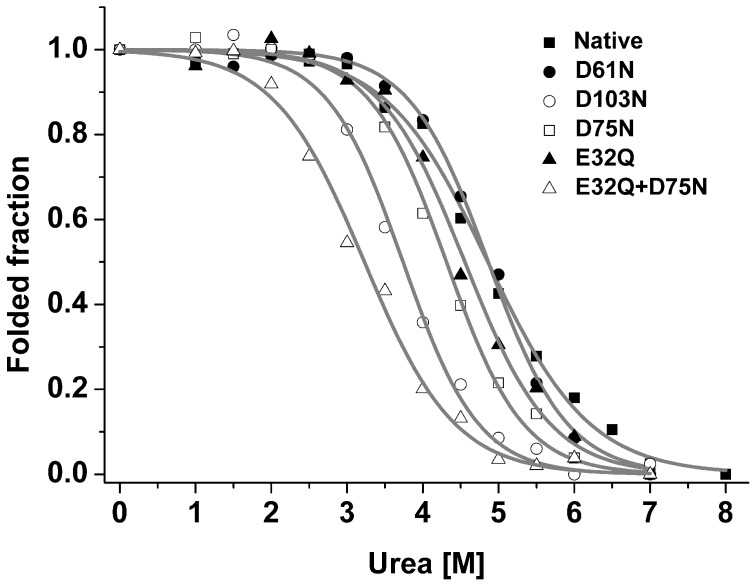
Urea-induced denaturation of CTD_Mi_ mutants. The solid lines represent the fitting curves.

To examine the importance of the solvent-exposed charges to the stability of CTD_Mi_, we prepared four conserved single-point mutants (E32Q, D61N, D75N and D103N) and one double-point mutant (E32Q/D75N). E32Q, D75N and D103N mutants showed significantly lower *C_m_* values than the wild type CTD_Mi_ although the mutation of D61N had only a slight effect on the stability. Moreover, double mutation reduced the *C_m_* from ∼4.8 M to ∼3.2 M urea (Figure 7, filled square and unfilled triangle). The results indicate that the solvent-exposed negatively charged residues are critical to the stability of CTD_Mi_. Interestingly, these negatively charged residues are conserved or partially conserved in all MiSp, but absent in the CTD_Ma_ of *A. diadematus* (Figure S1A). Besides the solvent-exposed negative charges, other factors such as hydrophobic interaction and hydrogen bonding which are slightly different in the two CTDs may also contribute to their significant difference in stability.

The chemical stability of LK-CTD_Mi_ and CTD_Mi_ was nearly identical (Figure 6A and 6B). This result shows that LK_Mi_ has no obvious effects on CTD_Mi_’s stability, implying that LK_Mi_ does not interact with CTD_Mi_ and confirming the conclusion drawn from the comparison of 2D HSQC spectra. The *T_m_* of LK-CTD_Mi_ was about 4°C lower than that of CTD_Mi_. This should not have resulted from the interaction of LK and CTD, but could be caused by the gradual slight aggregation of LK-CTD_Mi_ during the temperature ramping process. It is noteworthy that a small of amount of precipitate was observed only for LK-CTD_Mi_ and RP-LK-CTD_Mi_ in the thermal denaturation process. Similar to CTD_Mi_, LK-CTD_Mi_ was not influenced in stability by salt when NaCl concentration was below 500 mM.

RP_Mi_ also displayed a typical two-state unfolding profile, but showed a much lower stability than CTD_Mi_ (Figure 6C). Its *C_m_* went up from 1.4 M to 2.3 M urea when NaCl concentration was increased from 0 to 500 mM. This salt-dependent stability is similar to CTD_Ma_ but different from CTD_Mi_. Thermal denaturation (Figure S8, black and blue lines) also shows RP_Mi_ is much less stable than CTD_Mi_ (*T_m_*: 53 vs 71°C).

Unlike individual domains, the tri-domain fragment, RP-LK-CTD_Mi_, unfolded in two steps with the increase of urea concentrations. The denaturation curves were fitted (Figure 6D), and the extracted first and second *C_m_* values were the same as the *C_m_* values of the isolated RP_Mi_ and CTD_Mi_, respectively. This suggests RP_Mi_ tends to unfold first, followed by the unfolding of CTD_Mi_. The result also indicates that the three domains of RP-LK-CTD_Mi_ have no or very weak interactions in solution at low protein concentrations (<10 μM). This conclusion is consistent with that drawn from the NMR data analysis, and is further supported by the fact that the first-step unfolding of RP-LK-CTD_Mi_ is obviously dependent on salt (*C_m_*: 1.4–2.3 M) and the second-step unfolding is nearly independent of salt (*C_m_*: 4.7–4.9 M) (Figure 6D). The thermal denaturation data (Figure S8, cyan line) also indicates RP-LK-CTD_Mi_ unfolds in two steps in a non-cooperative way and the CTD in the tri-domain protein cannot stabilize the RP. Taken together, in spite of the high stability of CTD_Mi_, the tri-domain protein can easily undergo conformational changes due to the low stability of RP_Mi_ when the protein is in a dimeric structure. To achieve high stability, the tri-domain protein and full length MiSp may assemble to form high order structures like oligomers. Due to the absence of the RP domain in MaSp, MaSp and MiSp may use different mechanisms to achieve high stability.

### Solubility of CTD_Mi_, RP_Mi_, LK-CTD_Mi_ and RP-LK-CTD_Mi_

In 10 mM Tris buffer (pH 7.0), CTD_Mi_, LK-CTD_Mi_, RP-LK-CTD_Mi_, RP_Mi_ and RP-LK_Mi_ could be concentrated to about 300, 200, 150, 60 and 5 mg/ml before the observation of precipitate or gel. In 10 mM sodium phosphate (pH 7.0), the solubility of each protein was nearly the same as that in 10 mM Tris, indicating that the solubility is not affected by buffer. RP-LK_Mi_ had the lowest solubility and was prone to precipitate. Other domains or fragments did not precipitate during the concentration process, but they formed gel when their concentrations were above their corresponding maxima. As shown in Figure 2B, CTD_Mi_ is purely negatively charged and very polar on its surface. The electrostatic repulsion among negatively charged dimeric CTD_Mi_ can prevent self-assembly for the formation of random aggregates. Therefore, the high hydrophilicity and unique charge of CTD_Mi_ surface explains its extremely high solubility. On the basis of the high solubility and dimerization feature, CTD_Mi_ has been used to generate large sized silk-like proteins for strong silk fiber production [36].

Although RP_Mi_ was easy to form small oligomers, its water solubility was still quite high. This may be achieved by burying some solvent-exposed hydrophobic patches through formation of oligomeric structures. LK-RP_Mi_ was much less soluble than RP_Mi_, demonstrating that the solubility of LK_Mi_ should be significantly lower than 5 mg/ml. LK_Mi_’s low solubility agrees with its high hydrophobicity (Figure S7B). Interestingly, LK-CTD_Mi_ and RP-LK-CTD_Mi_ were very soluble. This may be explained by the presence of the highly soluble CTD_Mi_ through mutual compensation in solubility. Most likely, however, the high solubility of the LK-CTD_Mi_ and RP-LK-CTD_Mi_ is achieved through an alternative mechanism by forming oligomers. With this mechanism, the poorly soluble domains or fragments assemble to form oligomers through the aggregation-prone regions in LK_Mi_ or/and RP_Mi_, leading to partial burial of solvent-exposed hydrophobic regions and then resulting in high solubility of the entire protein. The presence of such oligomers in the sample of 3 mM RP-LK-CTD_Mi_ is evidenced by the observation of the significant increase of the line width of methyl proton NMR signals from the RP domain rather than from the CTD domain (Figure 4B). Similar to RP-LK-CTD_Mi_, the full length MiSp (which comprises about 15 repeats of RP-LK_Mi_) may also exist in oligomers in the silk gland where the protein concentrations can reach up to ∼50% w/w [35].

Our results also suggest that CTD_Mi_ and RP-LK_Mi_ play distinct roles in maintaining MiSp proteins in a highly water soluble form, i.e., RP-LK_Mi_ initiates the oligomerization through weak hydrophobic interactions among LK_Mi_ and RP_Mi_ domains and forms the core region of the oligomers, while CTD_Mi_ prevents MiSp from forming precipitate by staying outside the oligomer core. The structure of the oligomers formed by MiSp fragments seems quite different from that by TuSp fragments which resembles a micelle-like structure [22]. The different structures may result from the significant differences in the LK and CTD domains: LK_Mi_ (89aa) is much larger and more hydrophobic than the linker region between the RP and CTD of TuSp1 (48aa); the isolated CTD of TuSp exists as oligomers but CTD_Mi_ as dimers in aqueous solution [22]. MaSp was also proposed to form a micelle-like structure in which the repetitive domains are inside the micelle and CTD domains are outside [21]. Because of the significant difference in amino acid sequences, different types of silk proteins may use different ways to form high order structures for stable storage. In all the cases, however, CTDs are located outside the assembled structures to enhance the solubility of the assembled form. To fully understand why silk fibroins are highly soluble in silk glands, studies on the full length fibroins or large fragments including N- and C-terminal domains and several repetitive units are necessary.

### Stability against Shear Force

**Figure 8:**
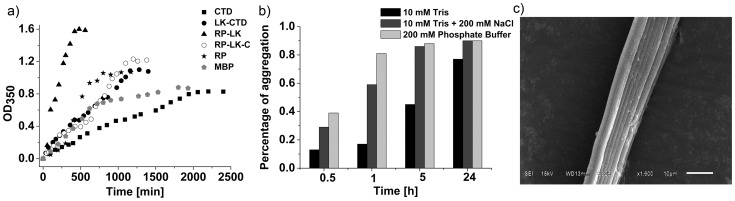
Shear-force-induced aggregation of MiSp fragments and MBP monitored by OD_350_ (a), dependence of aggregation of RP-LK-CTD_Mi_ on salts (b), and SEM of silk fibers formed by RP-LK-CTD_Mi_ (c). The scale bar is 10 µm.

In the natural silk spinning process, silk proteins pass through the spinneret in the silk gland and then become silk fibers. During this spinning, the proteins undergo conformational changes after encountering shear and elongational forces [3]. Here, we studied the effect of shear force on protein stability and aggregation by stirring protein solutions. In the absence of stirring (mechanical shear force), RP_Mi_, RP-LK_Mi_, RP-LK-CTD_Mi_, LK-CTD_Mi_, CTD_Mi_ and maltose binding protein (MBP) could maintain a soluble state under the condition of 0.05 mg/ml protein, 10 mM phosphate, pH 6.8 and 25°C without detectable precipitate within two days. In the presence of stirring, however, all of them tended to aggregate to form visible precipitate that is detectable at 350 nm. The changes in the amount of aggregated proteins with time are shown in Figure 8A. The *C_m_* and *T_m_* values of MBP in the absence of its ligand are 3.3 M urea and 63°C respectively [37], indicating MBP is more stable than RP_Mi_ but less stable than CTD_Mi_. Figure 8A shows that the aggregation rates of RP_Mi_, CTD_Mi_ and MBP are inversely proportional to their thermal or chemical stability. The aggregation should occur through partial protein unfolding and then assembly of the partially unfolded molecules. Note that the partially unfolded proteins have more solvent-exposed hydrophobic residues than the folded ones. Therefore the more stable a protein is, the slower the protein unfolding is, and the slower the shear-force-induced aggregation is.

The aggregation rates of CTD_Mi_ and RP_Mi_ were substantially accelerated by covalently linking LK_Mi_ domain to them respectively (Figure 8A). This result can be explained by the high aggregation propensity and low water solubility of the LK_Mi_ domain (Figure 3B and Figure S7B). Although LK_Mi_ is intrinsically disordered, stirring still greatly enhanced the aggregation rate of LK-CTD_Mi_ and RP-LK_Mi_, implying that the aggregation-prone regions are partially protected in the bi-domain protein fragments and shear force can reduce the protection. The protection may be achieved by partial local folding of the aggregation regions of LK_Mi_ or by the weak interaction between the disordered domain and folded domain. In any cases, LK_Mi_ plays a predominant role in the aggregation process of the protein fragments with an LK_Mi_ domain. Although RP-LK_Mi_, LK-CTD_Mi_ and RP-LK-CTD_Mi_ all contain an LK_Mi_ domain, RP-LK_Mi_ that is lacking a CTD_Mi_ displayed the highest aggregation rate. This result shows that CTD_Mi_ can slow down the aggregation rate and may play a role in regulating the assembly of silk protein molecules to form ordered structures.

Both NaCl and Na_3_PO_4_ were able to enhance the aggregation of RP-LK-CTD_Mi_ in the presence of shear force in similar rates (Figure 8B). In the case of MaSp, the effect of NaCl on the aggregation of CTD and RP-CTD was much less pronounced than that of Na_3_PO_4_ [21]. This result suggests that the fibroin storage and/or assembly conditions in MiSp and MaSp spider glands may be different.

### Fiber Formation

All the single and bi-domain constructs underwent nonspecific aggregation or precipitation in aqueous solution upon gentle shaking. Under the same condition, however, RP-LK-CTD_Mi_ could form small fibers with well-aligned structure and smooth surface even at a low protein concentration of ∼0.3 mg/ml (Figure 8C). The diameters of the formed fibers ranged from ∼2–10 µm, similar to that of the native MiSp silk [38]. The result reveals that all the three domains should participate in the fine-tuned process of fiber formation. Previous studies on MaSp and TuSp have shown that the minimum sequence requirement for a silk protein fragment to form silk fibers is that the fragment should contain a RP region and a terminal domain [20]–[22]. A recent study has revealed that the RP domain of aciniform spidroin alone could form silk fibers [39]. Therefore, MiSp fragments and full length MiSp may adopt a different fiber formation mechanism from other spider silk proteins.

Based on its low solubility (<<5 mg/ml), high hydrophobicity and aggregation propensity (Figure 3B, Figure S7B) and high aggregation rate in the presence of shear force (Figure 8A), LK_Mi_ may act as a nucleation site to initiate the assembly of RP-LK-CTD_Mi_ molecules through hydrophobic interactions among LK domains. Since RP_Mi_ is prone to form oligomers and is unstable against shear force and chemical and thermal denaturation, it may assist the LK domain to assemble silk protein molecules together and play a dominant role in conformational changes upon shear force. In the absence of CTD_Mi_, MiSp fragments such as RP-LK_Mi_ and RP_Mi_ formed only precipitate, indicating CTD_Mi_ is essential to silk fiber formation. The folded CTD_Mi_ may regulate the alignment of the assembled molecules by controlling the assembling rate since it can slow down the aggregation rate of RP-LK_Mi_ (Figure 8A), which leads to controlled formation of well-defined fibers rather than non-specific aggregation.

### Conclusions

CTD_Mi_, RP_Mi_ and LK_Mi_ have very distinct stability and solubility, which can be explained by their different structures, and each play specific roles in conferring the stable storage of MiSp fragments *in vitro* or full length MiSp in the silk gland. Due to the oligomerization-prone feature of RP_Mi_ and LK_Mi_, they are able to initiate the oligomerization through weak hydrophobic interactions among LK_Mi_ and RP_Mi_ domains and form the core region of the oligomers. On the other hand, because of the high solubility, CTD_Mi_ may prevent the MiSp fragments or full length MiSp from forming precipitate by staying outside the oligomer core.

Shear force greatly accelerates protein aggregation through protein partial unfolding. In the presence of shear force, the aggregation rate of a folded protein is inversely proportional to its thermal or chemical stability; while the aggregation rate of a multi-domain protein containing both folded and disordered domains is determined mainly by the property of the disordered domain and the solubility of the entire protein. Although all MiSp domains investigated here could self-assemble in the presence of shear force, only the tri-domain RP-LK-CTD_Mi_ formed well defined silk fibers, indicating that all three domains play distinct roles in fiber formation. According to our experimental data, we propose that the LK domain serves as a nucleation site to assemble different molecules together and CTD domains enable the arrangement of the assembled molecules in a highly ordered manner in the presence of shear force. Although MiSp, MaSp and TuSp fragments assemble in different ways, the relatively conserved CTD domains seem to play the same function, i.e., maintaining the assembled form in a highly soluble state before fiber formation and regulating the alignment of assembled molecules to form silk fibers. Due to the significant differences in biophysical properties among different types of CTDs and in primary structures and properties among different types of RPs, the molecular mechanisms of self-assembly and fiber formation for different types of silk proteins can be different. To reveal the detailed mechanisms, further studies on the structures of the assembled forms are required.

## Supporting Information

Figure S1 **Sequence alignments.** (a) C-terminal domains of MiSps from *Nephila antipodiana* (*N.a*), *Nephila clavipes* (*N.c*), *Latrodectus Hesperus* (*L.h*), *Lephilengys cruentata* (*L.c*) and *Uloborus diversus (U.d)* and MaSp from *Araneus diadematus* (*ADF-3*), (b) repetitive domains from *N.a, N.c, Nephilengys cruentata* (*N.c’*) and *Deinopis spinosa* (*D.s*).
(PDF)

Figure S2 **Sequence alignment of 5 types of linker domains from** ***Nephila antipodiana*** **.** Type 5 is the linker domain between the RP and CTD domains.
(PDF)

Figure S3 **Size exclusion chromatography profiles of CTD_Mi_ (12.2 kDa), LK-CTD_Mi_ (18.5 kDa), RP_Mi_ (14.5 kDa), RP-LK_Mi_ (20.8 kDa) and RP-LK-CTD_Mi_ (33 kDa).** Molecular weight makers are indicated on the top. Except for one RP_Mi_ (dashed curve) profile which was run in the presence of 100 mM NaCl, all other profiles were obtained under a buffer condition of 10 mM phosphate at pH 6.8.
(PDF)

Figure S4 **Temperature-induced denaturation of CTD_Mi_ and its mutant.** The curves were fitted using Eq. 1. All samples contained 10 µM protein and 10 mM phosphate at pH6.8.
(PDF)

Figure S5 **Comparison of surface plots of CTD_Ma_ (a) and CTD_Mi_ (b).** Hydrophobic residues are colored by a scale based on normalized hydrophobicity values: Phe (1.0) for yellow, Val (0.57) for light yellow and Gly (0.0) for white. Positively charged, negatively charged and polar residues (including all backbone and side-chain atoms) are colored by blue, red and light blue. Note that the exposed red and blue regions in the left panel are not from the charged carboxyl groups and guanidinium groups but from other parts of the charged residues.
(PDF)

Figure S6 **Comparison of hydrophobic interactions between α5 and α3 and between α5 and α1’ for CTD_Ma_ (a) and CTD_Mi_ (b).** Yellow and green represent hydrophobic and non-hydrophobic residues, respectively. Here Thr is considered as hydrophobic.
(PDF)

Figure S7 **Amino acid sequence of LK_Mi_ (a) and its hydrophobicity plot (b).**
(PDF)

Figure S8 **Temperature-induced unfolding of different MiSp fragments.** Except for RP-LK-CTD, the other curves were fitted using a two-state equation (Eq. 1). The curve for RP-LK-CTD was fitted using a linear combination of two two-state equations. All samples contained 10 µM protein and 10 mM phosphate buffer at pH 6.8.
(PDF)
