# Supplementary material for: Structural Characterization of Minor Ampullate Spidroin Domains and Their Distinct Roles in Fibroin Solubility and Fiber Formation
Source: PLoS One. 2013 Feb 13;8(2):e56142. doi: 10.1371/journal.pone.0056142 (PMC3571961; doi:10.1371/journal.pone.0056142)
Supplement: Figure S1 — Sequence alignments. (a) C-terminal domains of MiSps from Nephila antipodiana (N.a), Nephila clavipes (N.c), Latrodectus Hesperus (L.h), Lephilengys cruentata (L.c) and Uloborus diversus (U.d) and MaSp from Araneus diadematus (ADF-3), (b) repetitive domains from N.a, N.c, Nephilengys cruentata (N.c’) and Deinopis spinosa (D.s). (PDF) [file pone.0056142.s001.pdf]

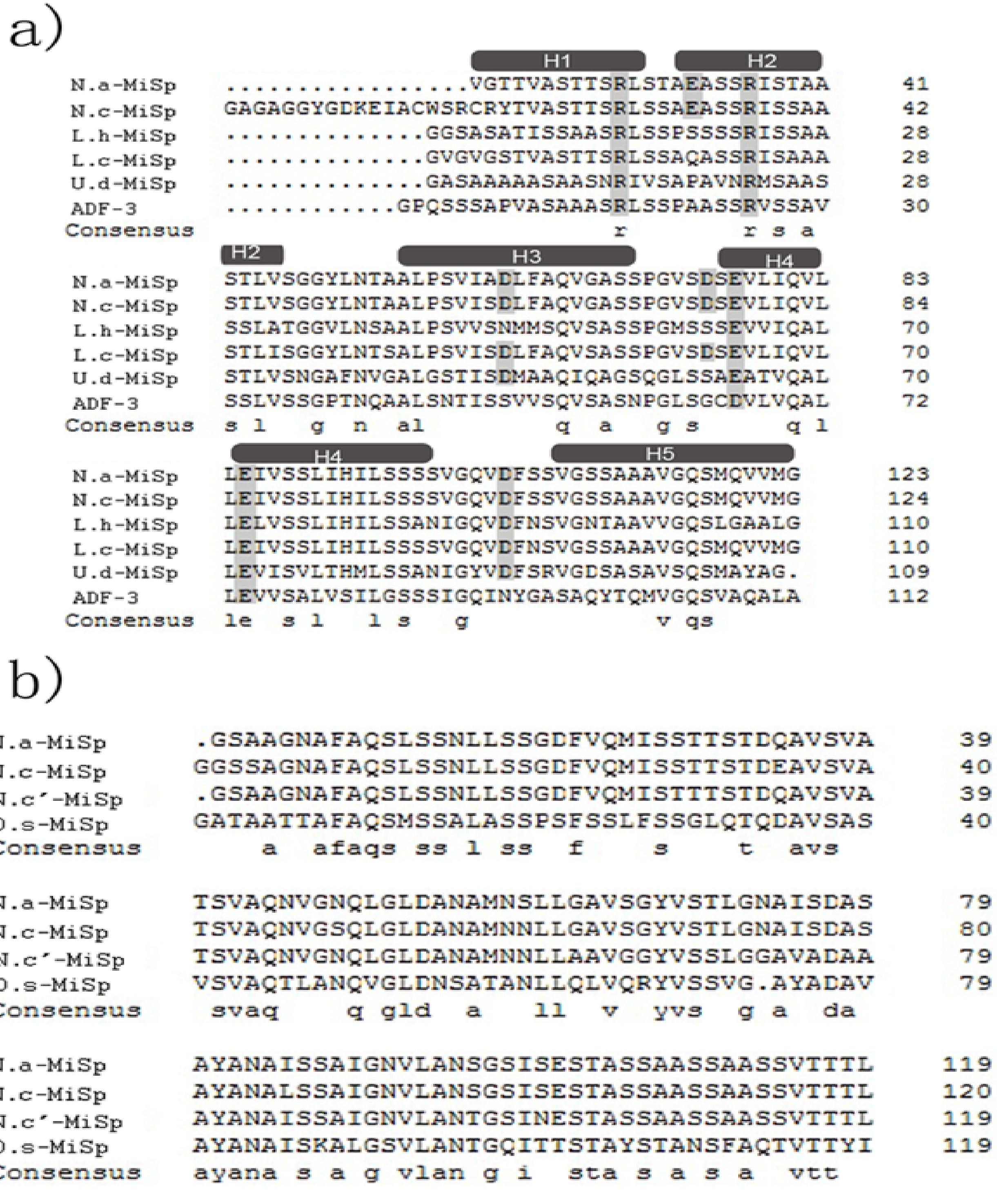

Figure S1. Sequence alignments of C-terminal domains (a) of MiSps from *Nephila antipodiana (N.a), Nephila clavipes (N.c), Latrodectus Hesperus (L.h), Lephilengys cruentata (L.c)* and *Uloborus diversus (U.d)* and MaSp from *Araneus diadematus (ADF-3)*, repetitive domains (b) from *N.a, N.c, Nephilengys cruentata (N.c')* and *Deinopis spinosa (D.s).*
