# Supplementary material for: Structural Characterization of Minor Ampullate Spidroin Domains and Their Distinct Roles in Fibroin Solubility and Fiber Formation
Source: PLoS One. 2013 Feb 13;8(2):e56142. doi: 10.1371/journal.pone.0056142 (PMC3571961; doi:10.1371/journal.pone.0056142)
Supplement: Figure S2 — Sequence alignment of 5 types of linker domains from Nephila antipodiana . Type 5 is the linker domain between the RP and CTD domains. (PDF) [file pone.0056142.s002.pdf]

```
1   AGGYGGLVGYGAGAAAAAAAGAGSGGAGGYGRGAGAGAGAAAGAGAGGAGGNGGQGGYGS 60
2   -GDYGGLVGY------------------------GAGAGAAAGAGAG------------- 22
3   -GDYGGLVGYG------------------------------------------------- 10
4   -GDYGGLVGYGAGAGAAAGAGAGAGRAGGYIGQGGYGAGAGVAAAAAAGAGAGATGGYGR 59
5   AGGYGGLVGYG------------------------------------------------- 11
     *.*******

1   GAGAAAAAGAGAGAGGAGGYGGQGGYGARAGAGAAAAAGAGAG-AGSY----GRGGGAGA 115
2   ---------------AAGGYGGQGGYGARAGAGAAAAAGAGAG-AGSY----GRGGGAGA 62
3   -----------------------------AGAGAAAAAGAGAG-AGSY----GRGAGAGA 36
4   GAGAGAAAAAGAGAGGAGGYGGQGGYGAGAGAGAAAAAGAGAG-AGSY----GRGGGAG- 113
5   -----------------------------AGAGAAAGAGAGAGGAGGYIGQGGYGAGAGA 42
                                 *******.****** **.*    * *.***

1   GAAAGAAAGGAGGY-RGQGGYGAGAGVAGAATSGAGAGGAGGYGRGAGAVAGAGAGSAAG 174
2   GAAAGAAAGGAGGY-RGQGGYGAGAGVAGAATSGAGAGGAGGYGRGAGAVAGAGAGSAAG 121
3   AAGAGAGAGGAGGY-RGQGGY------------GAGAGGAGGFGRGAGAGAGAGGGSAAG 83
4   ---AGAAAGGAGGY-RGQGGYGAGAGAAGAAAAGAGAGGAGGYGRGAGAGAGAGAGSAAG 169
5   AAAAGAGAGATGGYGRGAGAG---------ATNAGGYGGQGGYGAGARAFAGAGVG---- 89
       ***.**.:*** ** *.             ..* ** **:* ** * **** *
```

Figure S2. Sequence alignment of 5 types of linker domains from *Nephila antipodiana*. Type 5 is the linker domain between the RP and CTD domains.
