# Supplementary material for: Structural Characterization of Minor Ampullate Spidroin Domains and Their Distinct Roles in Fibroin Solubility and Fiber Formation
Source: PLoS One. 2013 Feb 13;8(2):e56142. doi: 10.1371/journal.pone.0056142 (PMC3571961; doi:10.1371/journal.pone.0056142)
Supplement: Figure S3 — Size exclusion chromatography profiles of CTDMi (12.2 kDa), LK-CTDMi (18.5 kDa), RPMi (14.5 kDa), RP-LKMi (20.8 kDa) and RP-LK-CTDMi (33 kDa). Molecular weight makers are indicated on the top. Except for one RPMi (dashed curve) profile which was run in the presence of 100 mM NaCl, all other profiles were obtained under a buffer condition of 10 mM phosphate at pH 6.8. (PDF) [file pone.0056142.s003.pdf]

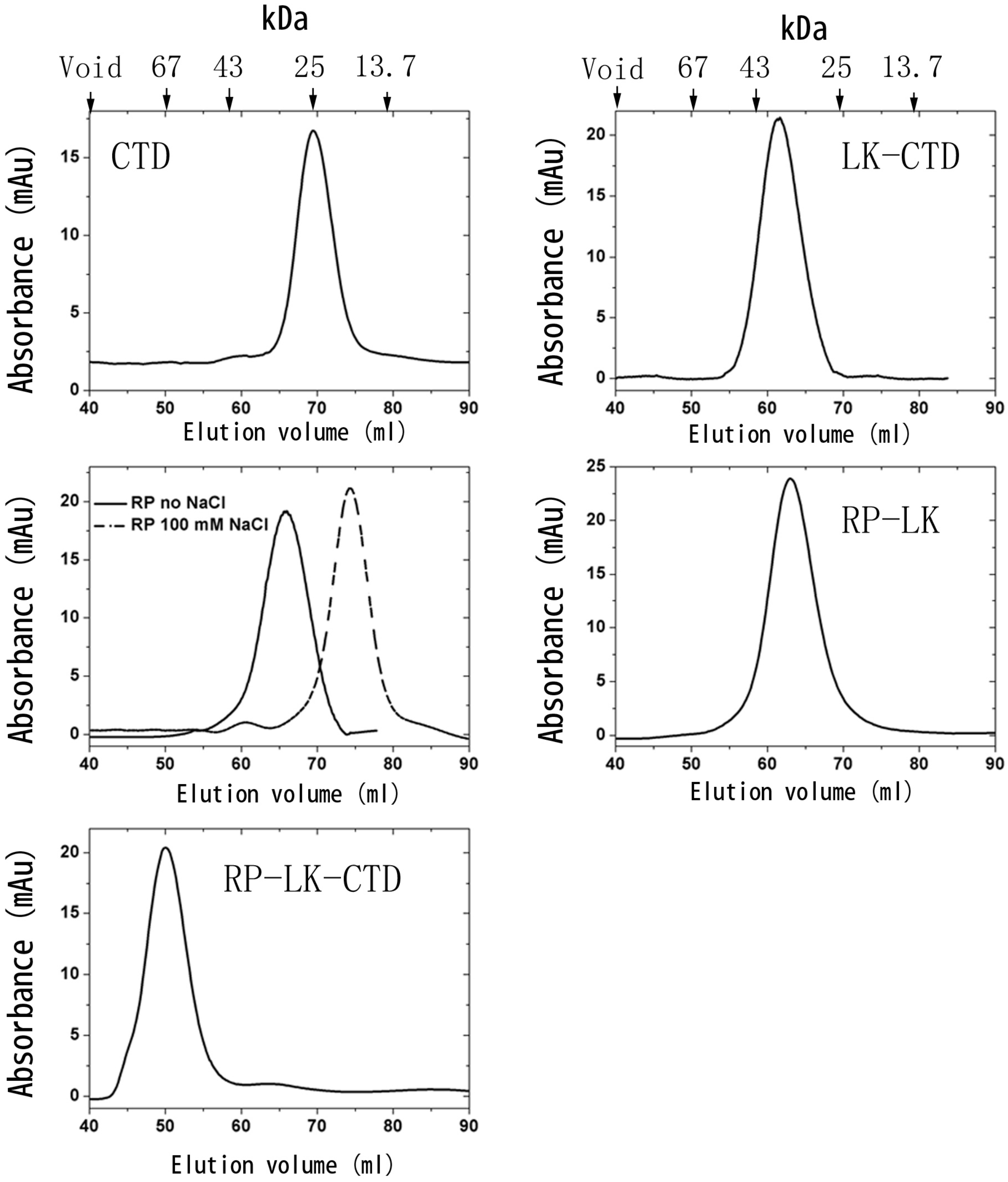

Figure S3. Size exclusion chromatography profiles of CTD_{Mi} (12.2 kDa), LK-CTD_{Mi} (18.5 kDa), RP_{Mi} (14.5 kDa), RP-LK_{Mi} (20.8 kDa), RP-LK-CTD_{Mi} (33 kDa). Molecular weight makers are indicated on the top. Except for one RP_{Mi} (dashed curve) profile which was run in the presence of 100 mM NaCl, all other profiles were obtained under a buffer condition of 10 mM phosphate at pH 6.8.
