# Supplementary material for: Structural Characterization of Minor Ampullate Spidroin Domains and Their Distinct Roles in Fibroin Solubility and Fiber Formation
Source: PLoS One. 2013 Feb 13;8(2):e56142. doi: 10.1371/journal.pone.0056142 (PMC3571961; doi:10.1371/journal.pone.0056142)
Supplement: Figure S4 — Temperature-induced denaturation of CTDMi and its mutant. The curves were fitted using Eq. 1. All samples contained 10 µM protein and 10 mM phosphate at pH6.8. (PDF) [file pone.0056142.s004.pdf]

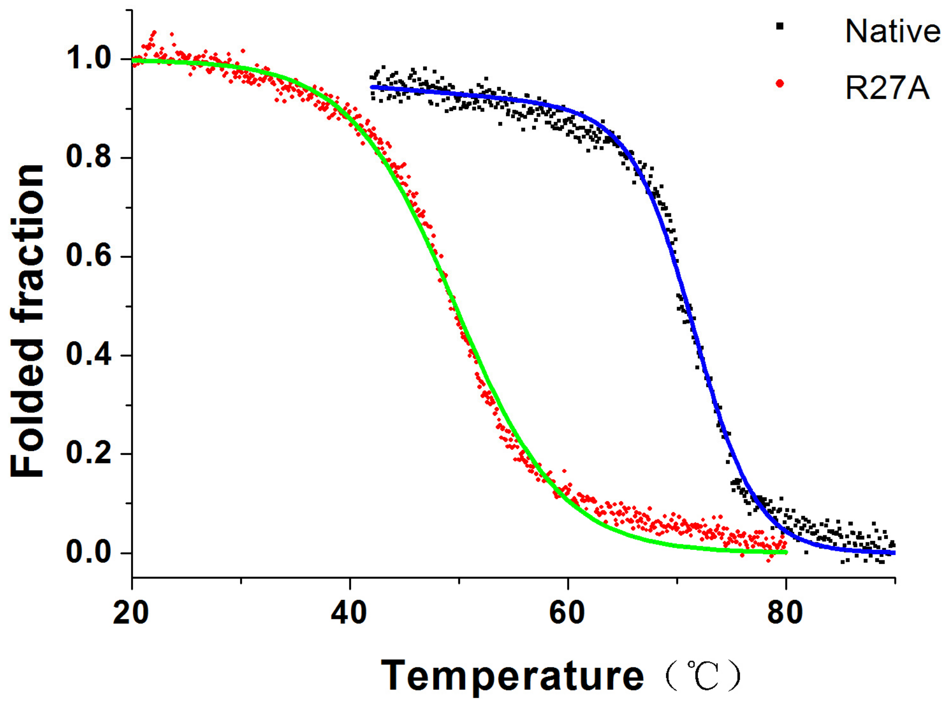


Figure S4. Temperature-induced denaturation of $CTD_{Mi}$ and its mutant. The curves were fitted using Eq. 1. All samples contained 10 μM protein and 10 mM phosphate at pH6.8.
