# Supplementary material for: Structural Characterization of Minor Ampullate Spidroin Domains and Their Distinct Roles in Fibroin Solubility and Fiber Formation
Source: PLoS One. 2013 Feb 13;8(2):e56142. doi: 10.1371/journal.pone.0056142 (PMC3571961; doi:10.1371/journal.pone.0056142)
Supplement: Figure S5 — Comparison of surface plots of CTDMa (a) and CTDMi (b). Hydrophobic residues are colored by a scale based on normalized hydrophobicity values: Phe (1.0) for yellow, Val (0.57) for light yellow and Gly (0.0) for white. Positively charged, negatively charged and polar residues (including all backbone and side-chain atoms) are colored by blue, red and light blue. Note that the exposed red and blue regions in the left panel are not from the charged carboxyl groups and guanidinium groups but from other parts of the charged residues. (PDF) [file pone.0056142.s005.pdf]

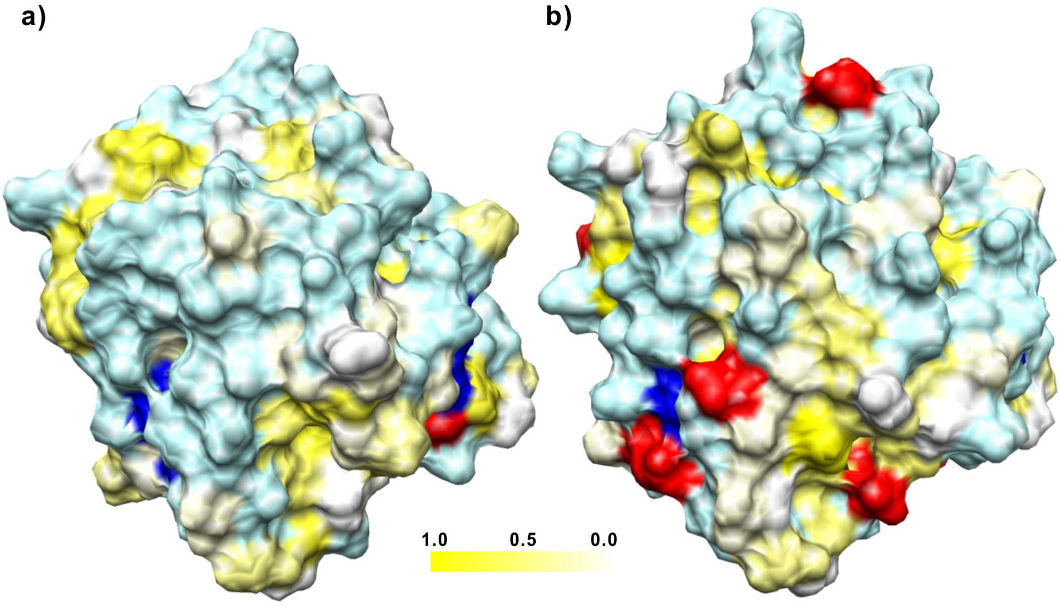

Figure S5. Comparison of surface plots of CTD_{Ma} (a) and CTD_{Mi} (b). Hydrophobic residues are colored by a scale based on normalized hydrophobicity values: Phe (1.0) for yellow, Val (0.57) for light yellow and Gly (0.0) for white. Positively charged, negatively charged and polar residues (including all backbone and side-chain atoms) are colored by blue, red and light blue. Note that the exposed red and blue regions in the left panel are not from the charged carboxyl groups and guanidinium groups but from other parts of the charged residues.
