# Supplementary material for: Structural Characterization of Minor Ampullate Spidroin Domains and Their Distinct Roles in Fibroin Solubility and Fiber Formation
Source: PLoS One. 2013 Feb 13;8(2):e56142. doi: 10.1371/journal.pone.0056142 (PMC3571961; doi:10.1371/journal.pone.0056142)
Supplement: Figure S6 — Comparison of hydrophobic interactions between α5 and α3 and between α5 and α1’ for CTDMa (a) and CTDMi (b). Yellow and green represent hydrophobic and non-hydrophobic residues, respectively. Here Thr is considered as hydrophobic. (PDF) [file pone.0056142.s006.pdf]

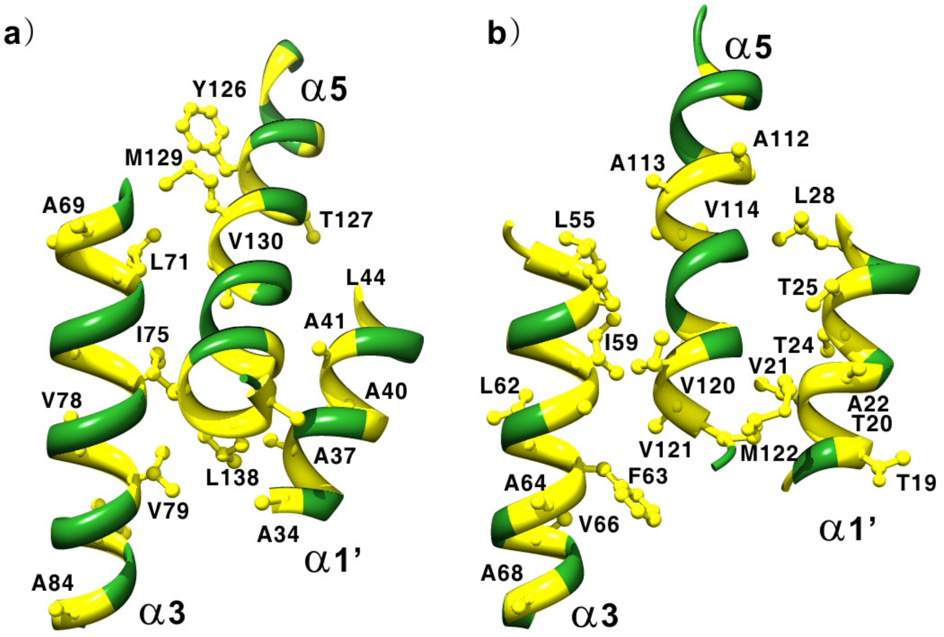


Figure S6. Comparison of hydrophobic interactions between α5 and α3 and between α5 and α1' for $CTD_{Ma}$ (a) and $CTD_{Mi}$ (b). Yellow and green represent hydrophobic and non-hydrophobic residues, respectively. Here Thr is considered as hydrophobic.
