# Supplementary material for: Structural Characterization of Minor Ampullate Spidroin Domains and Their Distinct Roles in Fibroin Solubility and Fiber Formation
Source: PLoS One. 2013 Feb 13;8(2):e56142. doi: 10.1371/journal.pone.0056142 (PMC3571961; doi:10.1371/journal.pone.0056142)
Supplement: Figure S7 — Amino acid sequence of LKMi (a) and its hydrophobicity plot (b). (PDF) [file pone.0056142.s007.pdf]

a

Amino acid sequence of linker domain
AGGYGGLVGY GAGAGAAAGA GAGAGGAGGY IGQGGYGAGA
GAAAAAGAGA GATGGYGRGA GAGATNAGGY GGQGGYGAGA
RAFAGAGVG

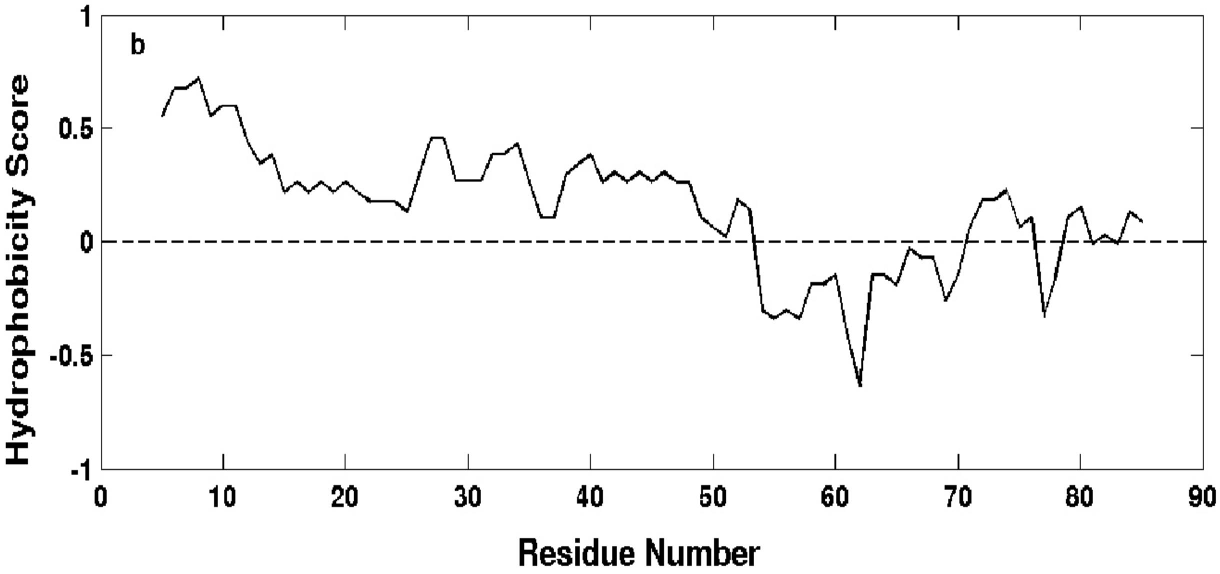

Figure S7. Amino acid sequence of LK_{Mi} (a) and its hydrophobicity plot (b).
