# Supplementary material for: Structural Characterization of Minor Ampullate Spidroin Domains and Their Distinct Roles in Fibroin Solubility and Fiber Formation
Source: PLoS One. 2013 Feb 13;8(2):e56142. doi: 10.1371/journal.pone.0056142 (PMC3571961; doi:10.1371/journal.pone.0056142)
Supplement: Figure S8 — Temperature-induced unfolding of different MiSp fragments. Except for RP-LK-CTD, the other curves were fitted using a two-state equation (Eq. 1). The curve for RP-LK-CTD was fitted using a linear combination of two two-state equations. All samples contained 10 µM protein and 10 mM phosphate buffer at pH 6.8. (PDF) [file pone.0056142.s008.pdf]

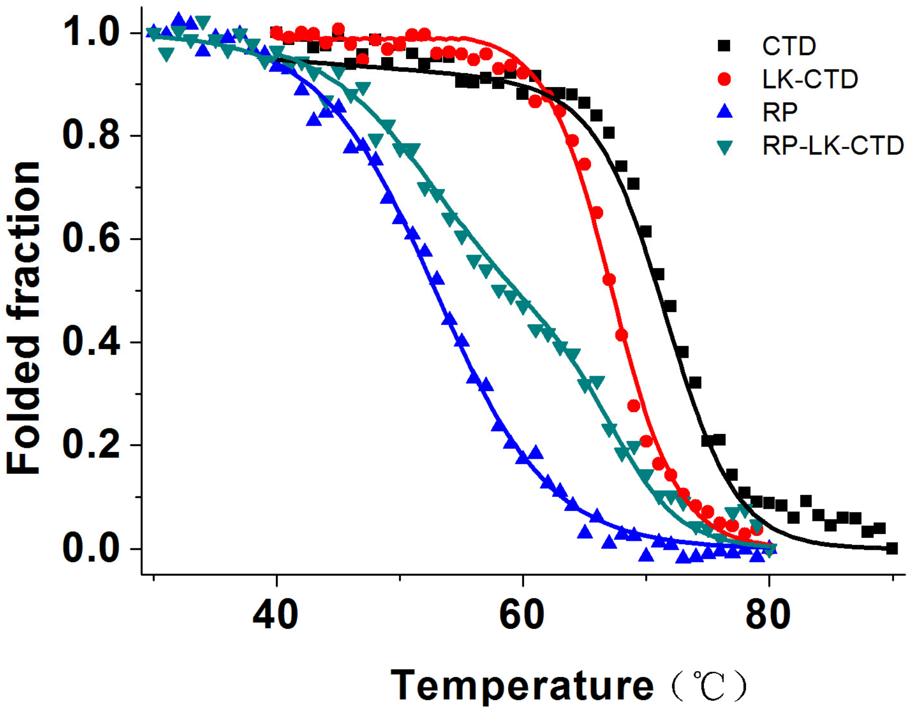

Figure S8. Temperature-induced unfolding of different MiSp fragments. Except for RP-LK-CTD, the other curves were fitted using a two-state equation (Eq. 1). The curve for RP-LK-CTD was fitted using a linear combination of two two-state equations. All samples contained 10 μM protein and 10 mM phosphate buffer at pH 6.8.
